# Molecular prevalence of *HBB*-associated hemoglobinopathy among reproductive-age adults and the prenatal diagnosis in Jiangxi Province, southern central China

**DOI:** 10.3389/fgene.2022.992073

**Published:** 2022-09-28

**Authors:** Haiyan Luo, Ting Huang, Qing Lu, Liuyang Zhang, Yonghua Xu, Yan Yang, Zhen Guo, Huizhen Yuan, Yinqin Shen, Shuhui Huang, Bicheng Yang, Yongyi Zou, Yanqiu Liu

**Affiliations:** ^1^ Department of Medical Genetics, Jiangxi Maternal and Child Health Hospital, Nanchang, Jiangxi, China; ^2^ Jiangxi Key Laboratory of Birth Defect Prevention and Control, Jiangxi Maternal and Child Health Hospital, Nanchang, Jiangxi, China; ^3^ Department of Ultrasound, Jiangxi Maternal and Child Health Hospital, Nanchang, Jiangxi, China

**Keywords:** HBB, hemoglobinopathy, mutation spectrum, prenatal diagnosis, central China-southern central China

## Abstract

**Background and aims:** Hemoglobinopathy associated with the *HBB* gene, with its two general subtypes as thalassemia and abnormal hemoglobin (Hb) variants, is one of the most prevalent hereditary Hb disorders worldwide. Herein we aimed to elucidate the prevalence of *ß*-thalassemia and abnormal hemoglobin variants and the prenatal diagnosis of the *HBB* gene in Jiangxi Province, southern central China.

**Methods:** Hematological indices and capillary Hb electrophoresis were conducted for 136,149 subjects who were admitted to Jiangxi Maternal and Child Health Hospital and requested for hemoglobinopathy investigation. Routine α- and *ß*-globin genotyping were performed by gap-polymerase chain reaction (Gap-PCR) and reverse dot-blot (RDB) hybridization for the 11,549 individuals suspected to be thalassemia carriers. For participants whose genotypes could not explain their hematological indices, further Sanger sequencing and Gap-PCR were conducted for the detection of rare or novel variants in related globin genes. Prenatal diagnosis was performed for 77 pregnant couples both carrying *ß*-thalassemia trait at appropriate gestational ages.

**Results:** Among the 11,549 subjects, 2,548 individuals were identified with *HBB*-associated hemoglobinopathy based on molecular analysis. A total of 2,358 subjects were identified as *ß*-thalassemia heterozygous carriers and nine cases were diagnosed as compound heterozygous *ß*-thalassemia. Additionally, 125 cases were detected with composite α- and *ß*-thalassemia and the remaining 56 individuals with abnormal Hb variants in the *HBB*. A total of 35 types of variants were identified in the *HBB* gene, including 26 types of *ß*-thalassemia and nine types of abnormal Hb variants. Four novel variants were firstly reported, including one variant in *HBA2* and three variants in *HBB*. Overall, 77 prenatal samples underwent *ß*-thalassemia molecular diagnosis; 20 fetuses were identified with normal *ß*-thalassemia genotypes, 30 fetuses as *ß*-thalassemia heterozygotes, 11 as homozygotes, and 16 as compound heterozygotes in *HBB*.

**Conclusion:** We have demonstrated a relatively high prevalence rate at 1.872% of *ß*-hemoglobinopathies including common and rare *ß*-thalassemia as well as abnormal Hb variants among large child-bearing population in the Jiangxi area of southern central China for the first time. Our data presents that prenatal diagnosis is an effective way to prevent and control birth defects of *ß*-thalassemia.

## Introduction

Hemoglobinopathies are one of the most common monogenic diseases worldwide (Ghosh et al., 2020). In the human body, hemoglobin (Hb) is a tetramer composed of two α-like and two *ß*-like globin chains, with the main function to deliver oxygen to the tissues (Ahmed et al., 2020). *ß*-Associated hemoglobinopathy is a group of hematological diseases caused by mutations in *ß*-globin genes, including *ß*-thalassemia and structural Hb variants (Kohne, 2011). *ß*-thalassemia (β-thal) is caused by reduced output of *ß*-globin chains, known as β^+^, or absent production of *ß*-globin chains, known as β^0^ (Origa, 2017). The altered production of normal *ß*-globin chain results in an imbalance of alpha/nonalpha chain synthesis, which is the major determinant of the clinical and hematological severity of *ß*-thal (Muncie and Campbell, 2009). The most common mutation of *ß*-thalassemia is small nucleotide variation (SNV), while a few others appear to be large deletions (Schwartz et al., 1988). The structural hemoglobin (Hb) variant, which leads to variable symptoms from almost asymptomatic to severe hemolytic anemia, is characterized by structural abnormalities caused by alteration of the globin peptide chain conformation in the *ß*-globin chains, which is typically based on single amino acid substitution resulting from a point mutation (Clarke and Higgins, 2000; Taher et al., 2018). Most individuals with heterozygous Hb variants exhibit normal hematological parameters but may display distinct Hb fractions by capillary electrophoresis (Keren et al., 2008; Altinier et al., 2013).

Despite a high prevalence in tropical and subtropical regions, *ß*-thal and structural Hb variants in *HBB* are now widely distributed worldwide because of population migration, and show abundant genetic diversity (Weatherall and Clegg, 2001). So far, there have been over 947 variants in *HBB* reported worldwide according to the online hemoglobin database (http://globin.bx.psu.edu/cgi-bin/hbvar/query_vars3). Furthermore, increasing reports on the prevalence and mutation spectrum of *ß*-hemoglobinopathy among different ethnic groups and different regions have improved the prevention of this major health problem in recent decades. China, as a country located in southeastern Asia, has high incidence of *ß*-hemoglobinopathy, especially for *ß*-thal in the southern provinces (Long et al., 2014). Many large-scale studies among the Chinese southern population, such as those from Hainan, Guangdong, Guangxi, and Fujian Province, have revealed the frequency and distribution of *ß*-thalassemia mutations in these specific geographic areas (Zhuang et al., 2020; Huang H. et al., 2021; Zhou et al., 2021; Wang et al., 2022). Several other studies have revealed the prevalence and mutation spectrum of abnormal Hb variants in some provinces of China (Lou et al., 2014; Huang et al., 2019; Xu A. et al., 2020). However, no previous study has focused on *ß*-hemoglobinopathy among large child-bearing age population in the Jiangxi area, a province located in southern central China next to Fujian and Guangdong Province.

Here, we explored the prevalence and mutation spectrum of *ß*-associated common and rare thalassemia and structural Hb variants among large-scale population of reproductive ages in Jiangxi Province, and provided prenatal diagnosis for those at risk of giving birth to a child with severe *ß*-thal intermediate or *ß*-thal major. Our work may provide a basis for genetic counseling, public education, and as a starting point to improve the prevention of *ß*-thal.

## Materials and methods

### Subjects

A total of 136,149 subjects of child-bearing age admitted to Jiangxi Maternal and Child Hospital for routine examination from January 2015 to December 2021 were enrolled in our study. The investigated individuals were mainly acquired from the Outpatient Department of the Assisted Reproductive Centre, Medical Genetics Centre, Obstetrics & Gynecology Department of our hospital. All of the included individuals came from rural or urban areas in Jiangxi Province and were aged from 18 to 50 years. This study was approved by the Ethics Committees of Jiangxi Maternal and Child Health Hospital, Jiangxi Province, China. All of the medical tests were performed after obtaining informed consent from the participants.

### Hematological studies and hemoglobin electrophoresis analysis

Conventional peripheral complete blood count (CBC) and capillary Hb electrophoresis were primarily screened for all the participants. Two EDTA-Na tubes with 2 ml of peripheral venous blood were obtained from each subject for routine blood examination and hemoglobin electrophoresis screening. Erythrocyte correlative indices were detected using a Sysmex XE-2100 blood analyzer (Sysmex Corporation) according to the standard operating procedure. The composition and content of hemoglobin was analyzed by a Sebia capillary electrophoresis system (Sebia, Inc.) according to the manufacturer’s instructions. Participants with erythrocyte indices with mean corpuscular volume (MCV) < 82 fL and (or) corpuscular hemoglobin (MCH) values <27 pg were taken as suspected thalassemia carriers. Subjects with hemoglobin A2 (HbA2) < 2.5% and HbA2 > 3.5% were suspected as probable α-thalassemia and *ß*-thalassemia carriers, respectively. Individuals screened with abnormal hemoglobin fraction or HbF values >5% were suspected as abnormal Hb variant or rare *ß*-thalassemia carriers.

### Routine thalassemia genetic analysis

A total of 11,549 subjects (female: 7,911, male: 3,638), including suspected thalassemia carriers and a few individuals who demanded genetic testing, underwent routine molecular detection for thalassemia. Firstly, 2 ml of peripheral venous blood was collected in an EDTA tube, and the genomic DNA of the peripheral venous blood was extracted using the GeneRotex Nucleic Acid Extraction System (GeneRotex, Xian, China). A NanoDrop spectrophotometer (Thermo Scientific, Wilmington, DE, United States) was used to qualify and quantify DNA. Gap-PCR and PCR-RDB (Yaneng BioSciences, Shenzhen) were used to detect three common α-thalassemia deletions (-SEA, -α3.7, and -α4.2) and three α-thalassemia point mutations (Hb Constant spring (Hb CS) (CD142, TAA > CAA), Hb Quong Sze (Hb QS) (CD125, CTG > CCG), and Hb Westmead (CD122, CAC > CAG), as well as 16 types of SNVs in *HBB*, including CD41-42 (-TCTT), CD43 (G > T), IVS-II-654 (C > T), CD17 (A > T), CD14-15 (+G), −28 (A > G), −29 (A > G), CD71-72 (+A), CD26 (G > A), IVS-I-1 (G > T), IVS-I-1 (G > A), CD27-28 (+C), IVS-I-5 (G > C), Cap+40–43 (-AAAC), initiation codon (ATG > AGG), and CD31 (-C).

### Rare and novel thalassemia genetic analyses

Gap-PCR was performed to detect six rare structural variations, including -THAI/, -α^21.9^/, -α^27.6^/, HK αα, anti4.2, *α* fusion gene for α-thal, and three rare *ß*-thal deletions, including Taiwanese, Gγ^+^(Aγδβ)^0^, and SEA-HPFH according to the manufacturer’s protocols (Yaneng BioSciences, Shenzhen, China). Sanger sequencing was used to detect the rare or novel SNVs in *HBA1*, *HBA2,* and *HBB*. The primers used in our study were listed in Table 1.

**TABLE 1:** Primers used for direct Sanger sequencing.

Primers	Sequence (5′→3′)	Length (bp)
HBA1-F	TGG AGG GTG GAG ACG TCC TG	20
HBA1-R	TCC ATC CCC TCC TCC CGC CCC TGC CTT TTC	30
HBA2-F	TGG AGG GTG GAG ACG TCC TG	20
HBA2-R	CCA TTG TTG GCA CAT TCC GG	20
HBB-F	AAC TCC TAA GCC AGT GCC AGA AGA GC	26
HBB-R	ATG CAC TGA CCT CCC ACA TTC CC	23

### Prenatal diagnosis

Chorionic villi sampling (CVS) in the first trimester, amniocentesis in the second trimester, and umbilical cord blood sampling in the third trimester were chosen based on the gestational age and willingness of the pregnant females. All of the fetal samples were collected from the pregnant mother under B-mode ultrasound-guidance at the appropriate gestational ages, with CVS from 12 to 14 weeks, amniocentesis from 18 to 26 weeks, and fetal blood sampling from 27 to 32 weeks. About 100 mg of villi tissue, 8 ml of amniotic fluid, or 2 ml of umbilical cord blood were collected. Extraction of fetal DNA was conducted using a QIAamp DNA Mini Kit (Qiagen, Germany) according to the manufacturer’s protocols. A NanoDrop spectrophotometer (Thermo Scientific, Wilmington, DE, United States) was used to qualify and quantify the DNA.

### Follow-up of pregnancy outcomes

The 77 couples were followed up by phone interview 1-year after prenatal diagnosis to record the outcomes of fetuses with severe thalassemia. Additionally, the phenotypes and development of fetuses with non-severe thalassemia were followed up 1 year after birth to validate the accuracy of the prenatal diagnosis. In our study, the mutant homozygote and double heterozygote were defined as severe forms of *ß*-thal.

### Statistical analysis

Statistical analysis was performed using SPSS statistical software version 20.0 (International Business Machines Corporation). Descriptive analysis was used to show the genotype and allele frequencies in different populations. The ratio of *ß*-thalassemia alleles, αβ-thalassemia, and abnormal Hb variants was calculated.

## Results

### Routine thalassemia genetic analysis and rare or novel thalassemia genetic analysis

Among the 11,549 subjects, 2,457 cases were detected with mutations by routine thalassemia testing, whereas the remaining 91 cases showed continuous hypochromic microcytic anemia, an increased HbA2 or HbF value, or an abnormal hemoglobin fraction by hematological screening and underwent further rare or novel thalassemia genetic analysis. Thirty individuals displaying an increased HbA2 value were identified with rare mutations in *HBB*, including CD54-58 (-TATGGGCAACCCT), -72 (T > A), codon 30 (AGG > AGC), -28(A > C), -31(A > G), SEA-HPFH, Taiwanese, IVS-I-6 (T > G), IVS-I-110 (G > A), and initiation codon ATG > GTG, with ten genotypes in all. Fifty-six individuals demonstrating abnormal hemoglobin fractions were identified as abnormal Hb variant carriers, including those with Hb J-Bangkok, Hb New York, Hb G-Taipei, Hb G-Coushatta, Hb D-Los Angeles, Hb Yaizu, Hb Deer Lodge, Hb E-Saskatoon, and Hb G-Siriraj, with a total of nine types. In addition, three individuals, all of whom showed normal HbA2 values and hematological parameters, were identified with three novel variants IVS-II-47 (G > C), IVS-II-300–308 (-A), IVS-II-806 (G > C) in *HBB*, separately. One individual were identified with the polymorphism IVS-II-81 C>T. Furthermore, one participant was identified with novel variant c.301-41_301-39delGGC in *HBA2* combined with the common *ß*-thal mutation IVS-II-654 (C > T).

### Overall population prevalence of *ß*-hemoglobinopathies in the jiangxi region

In our study, 11,549 individuals were suspected cases of *ß*-hemoglobinopathies by hematological indices or capillary analysis, and all of them underwent further genetic analysis. A total of 2,548 cases were diagnosed with *ß*-hemoglobinopathy, including 2,367 cases with *ß*-thal, 125 cases with composite α-and *ß*-thal, and 56 cases with abnormal Hb variants. The overall prevalence rate of *ß*-hemoglobinopathies in Jiangxi Province was 1.872% (2,548/13,6149), and the prevalence rates of *ß*-thal, coinheritance of αβ-thal and Hb variants were 1.739% (2,367/13,6149), 0.092% (125/13,6149), and 0.041% (56/13,6149), respectively. Carriers of composite α- and *ß*-thal and Hb variants were detected for the first time in a large population of child-bearing adults in Jiangxi and the detailed information was listed as in Table 2.

**TABLE 2:** Detailed constitution of *ß*-hemoglobinopathy among 13,6149 participants in the Jiangxi region.

Categories	Cases (n)	Ratio (%)
Heterozygous *ß*-thal carriers	2,358	1.732
Compound heterozygous *ß*-thal	9	0.007
Composite αβ-thal carriers	125	0.092
Abnormal Hb variants	56	0.041
Total	2,548	1.872

### Genotypes and mutation spectrum of *ß*-thal and structural Hb variants

We identified 83 genotypes in our cohort, with 34 of *ß*-thal, 39 of composite αβ-thal, and ten of abnormal Hb variants. Among the 2,367 subjects detected with *ß*-thal, we identified 26 kinds of *ß*-thal variants in all (Table.3). The most frequent genotype was β^IVS−II−654 (C>T)^/β^N^, and β^CD41/42(−TTCT)^/β^N^, with a remarkable proportion of 45.42% and 22.64%, respectively. The other common genotypes were β^CD17(A>T)^/β^N^, *ß* ^−28(A>G)^/β^N^, and β^CD27/28(+C)^/β^N^, with the corresponding proportions of 10.69%, 7.52%, and 4.86%. As for the allele frequency, the four most frequent mutations were IVS-II-654 (C>T), CD41/42 (-TTCT), CD17 (A>T), and −28 (A>G), with corresponding proportions of 41.9%, 27.5%, 12.6%, and 9.1%, which accounted for 91.1% of all *ß*-thal mutant alleles. A total of 125 subjects were detected as carriers of both α- and *ß*-globin variants (Table.4). Among them, 89.6% of genotypes consisted of common SNVs of the *ß*-globin gene combined with large deletions of the α-globin gene (–SEA/αα, -α3.7/αα, -α4.2/αα). Composite–SEA/αα and β^IVS−II−654 (C>T)^/β^N^ was the most common genotype. In our study, 56 subjects were identified with silent structural Hb variants in *HBB*, with ten genotypes in total. All of these individuals displayed normal hematological indices but abnormal hemoglobin fraction by capillary electrophoresis (Table.5). Hb J-Bangkok was the most prevalent Hb variant, accounting for 42.86%, followed by Hb New York and Hb Taipei. We also identified four rare Hb variants, including Hb Yaizu, Hb Deer Lodge, Hb E-Saskatoon, and Hb G-Siriraj. Furthermore, four novel variants were identified, including one variant c.301-41_301-39delGGC in *HBA2* and three variants (c.315 + 47G>C, c.315 + 300-308delA, and c.315-45G>C) in *HBB*. The hematological characteristics of the three subjects with novel variants were shown in Table 6.

**TABLE 3:** Genotype constitution of *ß*-thalassemia in Jiangxi Province.

Genotypes	HGVS nomenclature	Cases (n)	Ratio (%)
IVS-II-654 (C>T)	c.316–197C>T	1,075	45.42
Codons 41/42 (-TTCT)	c.126_129delCTTT	536	22.64
Codon 17 (A>T)	c.52A>T	253	10.69
-28 (A>G)	c.-78A>G	178	7.52
Codons 27/28 (+C)	c.84_85insC	115	4.86
Codons 71/72 (+A)	c.216_217insA	45	1.90
Hb E	c.79G>A	49	2.07
-29 (A>G)	c.-79A>G	30	1.27
Codon 43 (G>T)	c.130G>T	26	1.10
CD54-58 (-TATGGGCAACCCT)	c.165_177 del TATGGGCAACCCT	13	0.55
Codons 14/15 (+G)	c.45_46insG	11	0.46
-28 (A>C)	c.-78A>C	5	0.21
Cap+40–43 (-AAAC)	c.-11_-8delAAAC	4	0.17
-31 (A>G)	c.-81A>C	3	0.13
IVS-I-1 (G>T)	c.92 + 1G>T	2	0.08
IVS-II-81 (C>T)	c.315 + 81C>T	1	0.04
IVS-II-47 (G>C)	c.315 + 47 G > C	1	0.04
IVS-II-300–308 (-A)	c.315 + 300-308delA	1	0.04
IVS-II-806 (G- > C)	c.315-45G > C	1	0.04
-72 (T>A)	c.-122T>A	1	0.04
Codon 30 (AGG>AGC)	c.93G>C	1	0.04
IVS-I-6 (T>G)	c.92 + 6T>G	1	0.04
IVS-I-110 (G>A)	c.93-21G>A	1	0.04
Initiation codon ATG>GTG	c.1A>G	1	0.04
SEA-HPFH	g.5201647_5229059del	2	0.08
Taiwanese	g.69997_71353del1357	2	0.08
-28 (A>G)/IVS-II-81 (C>T)	—	1	0.04
Hb E/Hb E	—	2	0.08
-28 (A>G)/-28 (A>G)	—	1	0.04
IVS-II-654 (C>T)/CD27/28 (+C)	—	1	0.04
IVS-II-654 (C>T)/CD 41/42 (-TTCT)	—	1	0.04
CD 41/42 (-TTCT)/CD 27/28 (+C)	—	1	0.04
IVS-II-654 (C>T)/-28 (A>G)	—	1	0.04
Hb E/IVS-I-1 (G>T)	—	1	0.04
Total	—	2,367	100

**TABLE 4:** Genotypes of composite α- and *ß*-thalassemia in Jiangxi Region.

α-	β-	Cases (n)	Ratio (%)
-α^3.7^/αα	β^IVS−II−654 (C>T)^/β^N^	20	16.00
--SEA/αα		18	14.40
-α^4.2^/αα		3	2.40
α^CS^α/αα		3	2.40
α^WS^α/αα		2	1.60
α^QS^α/αα		2	1.60
α^c.301-41_301-39delGGC^α/αα		1	0.80
-α^3.7^/--SEA		1	0.80
--SEA/αα	β^Codons41/42 (−TTCT)^/β^N^	14	11.20
-α^3.7^/αα		13	10.40
-α^4.2^/αα		6	4.80
α^WS^α/αα		1	0.80
α^CS^α/αα		1	0.80
-α^4.2^/-α^4.2^		1	0.80
-α^3.7^/--SEA		1	0.80
α^QS^α/--SEA		1	0.80
--SEA/αα	β^Codon17(A>T)^/β^N^	3	2.40
-α^3.7^/αα		2	1.60
-α^4.2^/αα		2	1.60
α^WS^α/αα		1	0.80
α^CS^α/αα		1	0.80
--SEA/αα	β^−28 (A>G^)/β^N^	4	3.20
-α^3.7^/αα		3	2.40
-α^4.2^/αα		1	0.80
-α^3.7^/--SEA		1	0.80
-α^3.7^/αα	β^HbE^/β^N^	4	3.20
α^CS^α/αα		1	0.80
-α^3.7^/-α^3.7^		1	0.80
--SEA/αα	β^Codons27/28^ ^(+C)^/β^N^	2	1.60
-α^3.7^/αα		2	1.60
-α^3.7^/αα	β^HbE^/β ^HbE^	1	0.80
α^WS^α/-α^3.7^		1	0.80
--SEA/αα		1	0.80
-α^3.7^/αα	β^Codons71/72(+A)^/β^N^	1	0.80
--SEA/αα		1	0.80
--SEA/αα	β^−29 (A>G)^/β^N^	1	0.80
--SEA/αα	β^CAP+40–43 (−AAAC)^/β^N^	1	0.80
-α^3.7^/αα	β^IVS−I−1 (G>T)^/β^N^	1	0.80
--SEA/αα	β^Codons41/42(−TTCT)^/β^−28 (A>G)^	1	0.80
Total	—	125	100

**TABLE 5:** Abnormal hemoglobin variants in *HBB* and the hematological characteristics in this cohort in Jiangxi Province.

Hb variant	HGVS nomenclature	Hb (g/L)	MCV (fL)	MCH (pg)	Abnormal Hb concentration	Cases (n)	Ratio (%)
Hb J-Bangkok	c.170G>A (p.Gly57Asp)	128.6 ± 8.3	92.1 ± 1.8	29.9 ± 1.0	51.5 ± 0.6	24	42.86
Hb New York	c.341T>A (p.Val114Glu)	120.7 ± 10.1	89.5 ± 2.7	29.1 ± 0.5	43.9 ± 0.4	11	19.64
Hb G-Taipei	c.68A>G (p.Glu23Gly)	134.5 ± 7.5	86.4 ± 0.7	28.2 ± 0.6	39.5 ± 0.7	10	17.86
Hb G-Coushatta	c.68A>C (p.Glu23Ala)	140.7 ± 16.4	83.0 ± 0.9	30.0 ± 1.5	41.5 ± 0.7	3	5.36
Hb D-Los Angeles	c.364G>C (p.Glu122Gln)	137.0 ± 12.7	86.5 ± 2.3	28.9 ± 1.1	39.1 ± 1.5	3	5.36
Hb Yaizu	c.238G>A (p.Asp80Asn)	130	85.4	26.4	39.8	1	1.79
Hb Deer Lodge	c.8A>G (p His3Arg)	130	80.8	26.9	44.4	1	1.79
Hb E-Saskatoon	c.67G>A (p.Glu23Lys)	127	84.5	29.8	40.5	1	1.79
Hb G-Siriraj	c.22G>A (p.Glu8Lys)	123	85.2	30.3	30.6	1	1.79
Hb J-Bangkok and IVS-II-81 C>T	—	135	87.6	29.8	50.8	1	1.79
Total	—	—	—	—	—	56	100

**TABLE 6:** Hematological characteristics of the four subjects with novel variants.

Subject	Sex	Age (years)	Genotype	Hb (g/L)	MCV (fL)	MCH (pg)	HbA2 (%)	Ferritin (ng/ml)
No.1	Female	25	α^c.301-41_301-39delGGC^α/αα	107	60.4	19.1	5.5	60.0
			β^IVS−II−654 (C>T)^/β^N^					
No.2	Female	23	αα/αα	94	68.6	20.8	2.3	15.67
			β^IVS−II−47(G>C)^/β^N^					
No.3	Male	25	αα/αα	105	60.4	19.1	2.6	12.54
			β^IVS−II-300–308(−A)^/β^N^					
No.4	Female	23	αα/αα	98	72.1	26.8	3.2	—
			β^IVS−II−806(G>C)^/β^N^					

“/” indicates “unknown”; The reference ranges of ferritin are from 30 ng/ml ∼ 204 ng/ml.

### Prenatal diagnosis and postnatal follow-up outcomes

The screening strategy for pregnant or pre-pregnant couple in our study was recommended as follows: The female was genetically tested first, and if the female was determined to be a *ß*-thal carrier, her husband underwent further thalassemia genetic analysis. Prenatal diagnosis was recommended if both individuals in the couple were confirmed to be *ß*-thal trait carriers. In our study, 77 couples were identified as both being *ß*-thal carriers, and were considered to be at risk of having clinically affected offsprings. Prenatal diagnosis revealed 20 fetuses with normal genotypes in *HBB*, 26 fetuses as simple *ß*-thal carriers, four fetuses as composite α- and *ß*-thal carriers, 16 cases as compound heterozygous *ß*-thal patients, and 11 fetuses as homozygous *ß*-thal patients. The information on prenatal diagnosis was listed in detail in Supplementary Table S1.

No post-operative complications were found in 77 pregnant women with thalassemia, and 24 of the 77 pregnancies were terminated due to predicted severe *ß*-thal intermediate or major, including 10 with *ß*-thal homozygote and 14 with *ß*-thal double heterozygote. Following induced abortion, the fetal umbilical cord blood was sampled for genetic confirmation of *ß*-thal, which showed consistent results with prenatal diagnosis. All normal fetuses and those with *ß*-thal minor continued with the pregnancy. All of the pregnancy outcomes of the 77 families were listed in Supplementary Table S1. Telephone follow-up after the prenatal diagnosis revealed no thalassemia phenotypes in normal babies 6 months after birth, the babies with *ß*-thal minor had no or mild anemia symptoms and normal development milestones, while three babies with *ß*-thal double heterozygote demonstrated moderate anemia symptoms.

## Discussion

Here, we presented a large-scale genetic analysis of *HBB*-associated hemoglobinopathy among adults of child-bearing age in Jiangxi Province. The results showed a high prevalence of 1.872% for hemoglobinopathy of *ß*-globin in China, with the respective carrier rate at 1.739% for *ß*-thal, 0.092% for the composite αβ-thal, and 0.041% for the abnormal Hb variant. Based on previous studies, prevalence rate of *ß*-hemoglobinopathies among child-bearing population in typical geographic areas such as Guangxi (Xiong et al., 2010), Guangdong (Xu et al., 2004), Hubei, (Cheng et al., 2022), and Hunan Province (He et al., 2017) were listed in Table.7. Frequencies of *ß*-hemoglobinopathies in Jiangxi Province showed difference from other reports in China probably mainly due to geographic and ethnic background. The carrier rate of *ß*-thal and αβ-thalassemia in our study showed a slightly higher prevalence rate than the previously reported 1.82% in Jiangxi Province (Lin et al., 2014) mainly because of the wider geographic areas contained and more comprehensive of rare *ß*-thal in our study. Furthermore, the prevalence of abnormal Hb variants in *HBB* (0.041%) was determined among child-bearing adults in Jiangxi Province for the first time in our study, which was significantly lower than the reported 0.222% in Southeastern China (Huang et al., 2019; Xu L. et al., 2020) and the reported 0.185% for Hb variants in *HBB* in Dongguan Region of Guangdong Province (Lou et al., 2014). Notably, since Hb E is a special Hb variant with *ß*-thal hematological trait, we classified Hb E as *ß*-thal instead of abnormal Hb variant in our study, otherwise the carrier rate of abnormal Hb variant would have increased to 0.086% (116/136,149). We also observed some differences in the *ß*-thal mutation spectrum in Jiangxi Province compared to those of other provinces in China. For example, in contrast to Guangxi, Guangdong, and Hainan (Xu et al., 2004; Xiong et al., 2010; Wang et al., 2022), CD41/42 (-TCTT) is not the most prevalent mutation in Jiangxi Province, while compared with its surrounding provinces, such as Hunan and Hubei (Liu et al., 2019; Cheng et al., 2022), Jiangxi Province shares a more similar mutation spectrum, with IVS-II-654 (C>T), CD41/42 (-TCTT), and CD17 (A>T) as the three most frequent genotypes in these areas, accounting for almost 80% of all genotypes. Likewise, the spectrum of structural Hb variants in *HBB* in Jiangxi Province is similar to those reported in previous studies, with the most three common variants as Hb New York, Hb J-Bangkok, and Hb G-Taipei, while Hb YaiZu, Hb Deer Lodge, and Hb E-Saskatoon had been seldom reported previously in China.

**TABLE 7:** Comparison of *ß*-thal frequency among reproductive-age adults in different geographic areas in China.

References PMID	Geographic areas	Subjects (n)	Methods	Frequency of *ß*-thal	Frequency of αβ-thal
Zheng, 2021 34893129	Guilin, Guangxi Zhuang Autonomous Region	19,482	Gap-PCR + PCR-RDB	4.56% (889/19482)	0.63% (123/19,482)
Xu, 2020 15113860	Five regional centres in Guangdong Province	7,792	Gap-PCR + PCR-RDB	2.54% (198/7,792)	0.26% (20/7,792)
Wang, 2022 35119136	Hainan Province	166,936	Gap-PCR + PCR-RDB	1.38% (2,305/166,936)	1.18% (1,976/166,936)
Wang, 2021 34893128	Guiyang in Guizhou Province	4,572	PCR-RDB	1.99%	0.15%
				(91/4,572)	(7/4,572)
He, 2017 28674233	Changsha in Hunan Province	7,500	PCR-RDB	1.9%	0.08%
				(143/7,500)	(6/7,500)
Cheng, 2022 35244037	Eastern, Central, Western regions of Hubei Province	11,875	PCR-Flow cytometry	1.92%	0.03%
				(228/11875)	(3/11,875)
Lin, 2014 25000193	Three geographical areas of Jiangxi Province	9,489	PCR-RDB	1.70%	0.12%
				(161/9,489)	(11/9,489)
Li, 2020 32719015	Sichuan Province	42,155	Gap-PCR + PCR-RDB	0.75% (317/42,155)	0.045% (19/42,155)

β-thalassemia is clinically classified into four types, including *ß*-thal silent, *ß*-thal trait, *ß*-thal intermediate, and *ß*-thal major. *ß*-thalassemia intermediate, with the genotype of β^+^/β^+^ or β^+^/β^0^, has a more complex molecular basis than the other three types (Thein, 2013). Patients affected with *ß*-thal intermediate usually present with diverse phenotypes from mild-to-moderate anemia that does not require lifelong dependence on blood transfusion. In contrast, individuals with *ß*-thal major, with the genotype of β^+^/β^0^ or β^0^/β^0^ and severe anemia (Hb persistently <70 g/L), require regular blood transfusions and standardized iron-free therapy to survive (Writing Group For Practice Guidelines For Diagnosis And Treatment Of Genetic Diseases Medical Genetics Branch Of Chinese Medical Association et al., 2020). Cure of this disease depends on successful bone-marrow transplantation, which is expensive and has treatment-associated complications (Ali et al., 2021). Therefore, severe *ß*-thal intermediate or *ß*-thal major presents a major public health problem and a social burden in highly prevalent areas. For the prevention of this disease, prenatal diagnosis is the most useful method for a continuous decline in birth rates of babies with *ß*-thal major (Huang L. F. et al., 2021). Genotyping is strongly recommended for inclusion in the prenatal diagnosis of *ß*-thal, since precise prenatal diagnosis is crucial to prevent the development of thalassemia and for genetic counseling. Apart from prenatal diagnosis, pre-implantation genetic testing (PGT) for thalassemia has emerged as a new choice for some families, which, although expensive, causes less harm to the pregnant females.

Accurate genetic testing is the basis of effective birth defect prevention of thalassemia in pre-pregnancy or prenatal genetic counseling. Although traditional genetic testing is a low-cost and time-saving strategy, it only focuses on the most common regional mutations, which may lead to missed diagnosis of rare or novel variants. Therefore, further supplementary molecular testing is required for individuals whose genotypes cannot explain the hematological indices in clinical practice. In our study, 30 participants with increased HbA2 values and microcytic hypochromic hematological profiles were identified with rare *ß*-thal mutations. Overall, 10 types of rare *ß*-thal mutations that had not been previously reported in Jiangxi Province were identified, representing 1.18% (30/2,548) of all the mutations, with IVS-I-6 (T>G) identified for the first time worldwide. Among the 30 cases with rare *ß*-thal mutations, the most common genotype was CD54-58 (-TATGGGCAACCCT), followed by -28 (A>C), suggesting that these two mutations show a more frequent carrier rate than some of the 17 point mutations tested by traditional PCR-RDB method. In addition, we also identified four novel variants, including *HBA2*:c.301-41_301-39delGGC, *HBB*:IVS-II-47 (G>C), IVS-II-300–308 (-A), and IVS-II-806 (G>C), even though their pathogenesis still requires further study. Notably, we found that participants with the variant β^+^ Cap+40–43 (-AAAC) identified by traditional thalassemia genetic testing, did not demonstrate the hematological characteristics of increased HbA2 values or microcytic hypochromic when iron-deficiency is excluded. As reported by Li, the pathogenicity of this variant remains uncertain and may be regarded as a silent *ß*-thal allele (Zhao et al., 2020). Moreover, based on our findings, we propose that the two frequent mutations CD54-58 (-TATGGGCAACCCT) and -28 (A>C) should be included into traditional genetic testing based on specific geographic background in Jiangxi Province. Secondly, hematological indices of CBC test and hemoglobin electrophoresis are both indispensable for the correct recognition of *ß*-hemoglobinopathies. Finally, more comprehensive and efficient molecular testing approaches may be considered for identification of both common and rare variants of thalassemia simultaneously.

Despite these strengths, certain limitations of our study should also be acknowledged. As mutations in *HBD* can cover the typical increased HbA2 value for individuals with *ß*-thal (Chen et al., 2020), missed diagnosis may occur based on the current hematological screening strategy as the hematological parameters might be normal or borderline indices for some rare thalassemia mutation types or Hb variants. Thus, it is possible that silent mutations in α-globin genes, *α* triplications, or Hb variants without abnormal Hb composition were missed in this study. In recent years, the combined Gap-PCR and next-generation sequencing (NGS) has emerged and used as an alternative more complete and versatile molecular approach for thalassemia genetic testing (Shang et al., 2017), even though it is considered scalable, cost-effective, and comprehensive. Besides, another alternative efficient, accurate, and cost-effective single platform technology generating longer PCR fragments including intergenic and intragenic regions to identify other rare variants undetectable by the typical panel test have been studied and validated (Xu L. et al., 2020). Studies revealed that this new emerging methods with universal approach to comprehensive analysis of variants in homologous genes *HBA1*/*HBA2*, cis or trans configuration, and complex structural rearrangements are hardly associated with false-negative and false-positive results (Liang et al., 2021).

## Conclusion

In summary, we have provided the most comprehensive mutation spectrum of thalassemia and abnormal Hb variants mutation spectrum in the *HBB* gene among adults of reproductive-age in Jiangxi Province to date. We also provided intensive genetic counseling and prenatal diagnosis for couples with indications to avoid the birth of fetuses with *ß*-thal intermediate or major. Our work may provide as a baseline for genetic counseling, public education, and as a reference to improve the prevention of *ß*-thal.

## Data Availability

The original contributions presented in the study are included in the article/Supplementary Material, further inquiries can be directed to the corresponding author.
