# Supplementary material for: Molecular prevalence of HBB-associated hemoglobinopathy among reproductive-age adults and the prenatal diagnosis in Jiangxi Province, southern central China
Source: Front Genet. 2022 Sep 28;13:992073. doi: 10.3389/fgene.2022.992073 (PMC9554255; doi:10.3389/fgene.2022.992073)
Supplement: Supplementary file 1 [file Table1.pdf]

Supplementary data

Table.S1 Detailed information and outcomes of prenatal diagnosis of β-thal in the 77 families

Father	Mother	Fetus	Numbers (n)	Pregnancy outcome
αα/αα, $\beta^{N}\beta^{654}$	αα/αα, $\beta^{N}\beta^{654}$	αα/αα, $\beta^{N}\beta^{N}$	3	Continued
αα/αα, $\beta^{N}\beta^{654}$	αα/αα, $\beta^{N}\beta^{654}$	αα/αα, $\beta^{N}\beta^{654}$	6	Continued
αα/αα, $\beta^{N}\beta^{654}$	αα/αα, $\beta^{N}\beta^{654}$	αα/αα, $\beta^{654}\beta^{654}$	8	Terminated
αα/αα, $\beta^{N}\beta^{654}$	αα/αα, $\beta^{N}\beta^{CD17}$	αα/αα, $\beta^{N}\beta^{N}$	2	Continued
αα/αα, $\beta^{N}\beta^{654}$	αα/αα, $\beta^{N}\beta^{CD17}$	αα/αα, $\beta^{N}\beta^{654}$	2	Continued
αα/αα,$\beta^{N}\beta^{654}$	αα/αα, $\beta^{N}\beta^{CD41\text{-}42}$	αα/αα, $\beta^{N}\beta^{N}$	1	Continued
αα/αα, $\beta^{N}\beta^{654}$	αα/αα, $\beta^{N}\beta^{CD41\text{-}42}$	αα/αα, $\beta^{N}\beta^{CD41\text{-}42}$	4	Continued
αα/αα, $\beta^{N}\beta^{654}$	αα/αα, $\beta^{N}\beta^{CD41\text{-}42}$	αα/αα, $\beta^{654}\beta^{CD41\text{-}42}$	2	Terminated
αα/αα, $\beta^{N}\beta^{654}$	αα/αα, $\beta^{N}\beta^{-28}$	αα/αα, $\beta^{N}\beta^{N}$	1	Continued
αα/αα, $\beta^{N}\beta^{654}$	αα/αα, $\beta^{N}\beta^{-28}$	αα/αα, $\beta^{654}\beta^{-28}$	2	Terminated
αα/αα, $\beta^{N}\beta^{654}$	αα/αα, $\beta^{N}\beta^{CD27/28}$	αα/αα, $\beta^{654}\beta^{CD27/28}$	1	Terminated
αα/αα, $\beta^{N}\beta^{CD41\text{-}42}$	αα/αα, $\beta^{N}\beta^{CD41\text{-}42}$	αα/αα, $\beta^{N}\beta^{N}$	3	Continued
αα/αα, $\beta^{N}\beta^{CD41\text{-}42}$	αα/αα, $\beta^{N}\beta^{CD41\text{-}42}$	αα/αα, $\beta^{CD41\text{-}42}\beta^{CD41\text{-}42}$	2	Terminated
αα/αα, $\beta^{N}\beta^{CD41\text{-}42}$	αα/αα, $\beta^{N}\beta^{654}$	αα/αα, $\beta^{N}\beta^{N}$	4	Continued
αα/αα,$\beta^{N}\beta^{CD41\text{-}42}$	αα/αα, $\beta^{N}\beta^{654}$	αα/αα, $\beta^{654}\beta^{CD41\text{-}42}$	2	Terminated
αα/αα, $\beta^{N}\beta^{CD41\text{-}42}$	αα/αα, $\beta^{N}\beta^{-28}$	αα/αα, $\beta^{N}\beta^{N}$	1	Continued
αα/αα, $\beta^{N}\beta^{CD41\text{-}42}$	αα/αα, $\beta^{N}\beta^{-29}$	αα/αα, $\beta^{N}\beta^{CD41\text{-}42}$	1	Continued
αα/αα, $\beta^{N}\beta^{CD41\text{-}42}$	αα/αα, $\beta^{N}\beta^{CD14\text{-}15}$	αα/αα, $\beta^{N}\beta^{CD14\text{-}15}$	1	Continued
αα/αα, $\beta^{N}\beta^{CD41\text{-}42}$	αα/αα, $\beta^{N}\beta^{CD17}$	αα/αα, $\beta^{N}\beta^{CD41\text{-}42}$	1	Continued
αα/αα, $\beta^{N}\beta^{CD41\text{-}42}$	αα/αα, $\beta^{N}\beta^{CD71\text{-}72}$	αα/αα, $\beta^{CD41\text{-}42}\beta^{CD71\text{-}72}$	1	Terminated
αα/αα, $\beta^{N}\beta^{-28}$	αα/αα, $\beta^{N}\beta^{654}$	αα/αα, $\beta^{N}\beta^{N}$	1	Continued
αα/αα, $\beta^{N}\beta^{-28}$	αα/αα, $\beta^{N}\beta^{654}$	αα/αα, $\beta^{N}\beta^{-28}$	1	Continued
αα/αα, $\beta^{N}\beta^{-28}$	αα/αα, $\beta^{N}\beta^{654}$	αα/αα, $\beta^{654}\beta^{-28}$	2	Terminated
αα/αα, $\beta^{N}\beta^{-28}$	αα/αα, $\beta^{N}\beta^{-28}$	αα/αα, $\beta^{N}\beta^{-28}$	1	Continued
αα/αα, $\beta^{N}\beta^{-28}$	αα/αα, $\beta^{N}\beta^{-28}$	αα/αα, $\beta^{-28}\beta^{-28}$	1	Continued
αα/αα, $\beta^{N}\beta^{-28}$	αα/αα, $\beta^{N}\beta^{CD17}$	αα/αα, $\beta^{-28}\beta^{CD17}$	1	Terminated
αα/αα, $\beta^{N}\beta^{-28}$	αα/αα, $\beta^{N}\beta^{CD27/28}$	αα/αα, $\beta^{N}\beta^{CD27/28}$	1	Continued
αα/αα, $\beta^{N}\beta^{-28}$	αα/αα, $\beta^{N}\beta^{CD41\text{-}42}$	αα/αα, $\beta^{-28}\beta^{CD41\text{-}42}$	1	Terminated
αα/αα, $\beta^{N}\beta^{CD27/28}$	αα/αα, $\beta^{N}\beta^{654}$	αα/αα, $\beta^{N}\beta^{N}$	1	Continued
αα/αα, $\beta^{N}\beta^{CD27/28}$	αα/αα, $\beta^{N}\beta^{CD17}$	αα/αα, $\beta^{N}\beta^{CD17}$	1	Continued
αα/αα, $\beta^{N}\beta^{CD17}$	αα/αα, $\beta^{N}\beta^{654}$	αα/αα, $\beta^{N}\beta^{N}$	1	Continued
αα/αα, $\beta^{N}\beta^{CD17}$	αα/αα, $\beta^{N}\beta^{654}$	αα/αα, $\beta^{N}\beta^{654}$	1	Continued
αα/αα, $\beta^{N}\beta^{CD17}$	αα/αα, $\beta^{N}\beta^{654}$	αα/αα, $\beta^{N}\beta^{CD17}$	1	Continued
αα/αα, $\beta^{N}\beta^{CD17}$	αα/αα, $\beta^{N}\beta^{-28}$	αα/αα, $\beta^{N}\beta^{CD17}$	2	Continued
αα/αα, $\beta^{N}\beta^{CD17}$	αα/αα, $\beta^{N}\beta^{-28}$	αα/αα, $\beta^{-28}\beta^{CD17}$	1	Terminated
αα/αα, $\beta^{N}\beta^{CD17}$	αα/αα, $\beta^{N}\beta^{CD14\text{-}15}$	αα/αα, $\beta^{N}\beta^{CD17}$	1	Continued
αα/αα, $\beta^{N}\beta^{CD17}$	αα/αα, $\beta^{N}\beta^{CD17}$	αα/αα, $\beta^{N}\beta^{CD17}$	1	Continued
αα/αα, $\beta^{N}\beta^{CD17}$	αα/αα, $\beta^{N}\beta^{CD41\text{-}42}$	αα/αα, $\beta^{N}\beta^{CD41\text{-}42}$	1	Continued
αα/αα, $\beta^{N}\beta^{-29}$	αα/αα, $\beta^{N}\beta^{-28}$	αα/αα, $\beta^{-29}\beta^{-28}$	1	Continued
αα/αα, $\beta^{N}\beta^{E}$	αα/αα, $\beta^{N}\beta^{-29}$	αα/αα, $\beta^{-29}\beta^{E}$	1	Continued
αα/--SEA, $\beta^{N}\beta^{-28}$	αα/αα, $\beta^{N}\beta^{654}$	αα/--SEA, $\beta^{N}\beta^{654}$	1	Continued

αα/--SEA, $β^{N}β^{-28}$	αα/αα, $β^{N}β^{654}$	αα/--SEA, $β^{N}β^{-28}$	1	Continued
αα/-α3.7, $β^{N}β^{-28}$	αα/αα, $β^{N}β^{654}$	αα/αα, $β^{N}β^{N}$	1	Continued
αα/-α4.2, $β^{N}β^{654}$	αα/--SEA, $β^{N}β^{654}$	-α4.2/--SEA, $β^{N}β^{654}$	1	Continued
αα/-α3.7, $β^{N}β^{654}$	αα/αα, $β^{N}β^{-28}$	αα/-α3.7, $β^{N}β^{N}$	1	Continued
αα/αα, $β^{N}β^{CD41-42}$	αα/αα, $β^{N}β^{CD27/28}$	αα/-α3.7, $β^{CD41-42}/β^{CD27/28}$	1	Terminated
αα/αα, $β^{N}β^{CD27/28}$	-α3.7/--SEA, $β^{N}β^{654}$	αα/-α3.7, $β^{N}β^{654}$	1	Continued
**Total**	/	/	**77**	/

Notes: $β^{-28}$ indicates the mutation -28 (A>G).
